# Clustering Molecular Dynamics Trajectories for Optimizing Docking Experiments

**DOI:** 10.1155/2015/916240

**Published:** 2015-03-22

**Authors:** Renata De Paris, Christian V. Quevedo, Duncan D. Ruiz, Osmar Norberto de Souza, Rodrigo C. Barros

**Affiliations:** ^1^Grupo de Pesquisa em Aprendizado de Máquina e Inteligência de Negócio (GPIN), Faculdade de Informática, PUCRS, Prédio 32, Sala 628, 90619-900 Porto Alegre, RS, Brazil; ^2^Laboratório de Bioinformática, Modelagem e Simulação de Biossistemas (LABIO), Faculdade de Informática, PUCRS, Prédio 32, Sala 602, 90619-900 Porto Alegre, RS, Brazil

## Abstract

Molecular dynamics simulations of protein receptors have become an attractive tool for rational drug discovery. However, the high computational cost of employing molecular dynamics trajectories in virtual screening of large repositories threats the feasibility of this task. Computational intelligence techniques have been applied in this context, with the ultimate goal of reducing the overall computational cost so the task can become feasible. Particularly, clustering algorithms have been widely used as a means to reduce the dimensionality of molecular dynamics trajectories. In this paper, we develop a novel methodology for clustering entire trajectories using structural features from the substrate-binding cavity of the receptor in order to optimize docking experiments on a cloud-based environment. The resulting partition was selected based on three clustering validity criteria, and it was further validated by analyzing the interactions between 20 ligands and a fully flexible receptor (FFR) model containing a 20 ns molecular dynamics simulation trajectory. Our proposed methodology shows that taking into account features of the substrate-binding cavity as input for the *k*-means algorithm is a promising technique for accurately selecting ensembles of representative structures tailored to a specific ligand.

## 1. Introduction

Proteins are intrinsically flexible systems and this flexibility is relevant to determine their functions for discovering new potential drugs [1]. Nevertheless, realistic docking softwares that consider the molecular flexibility for both receptor and ligand are still far from accurately and reliably predicting complex structures for arbitrary ligand-receptor pairs [2]. Molecular dynamics (MD) simulations are a well-known technique used to investigate the detailed, atomic dynamic behavior of proteins in aqueous solution. It is capable of recognizing subtle internal motions and slow conformational changes, including bond vibration, chain reorientation, and backbone rearrangements at different timescales [3, 4].

Even though MD simulation is one of the most versatile and widely applied approaches to represent the natural behavior of ligand and protein within a flexible environment, it is also considered a time-consuming process. The high computational cost further increases when docking experiments are used for the fast screening of large virtual libraries against an entire MD ensemble which is applied to exploit all conformations of the protein receptor [3].

In this paper, an MD ensemble is called a fully flexible receptor (FFR) model [5], which typically has over 10^4^ MD structures. For this reason, recent studies on combining docking and MD simulations have created novel techniques to systematically reduce the number of MD structures without losing essential structural/dynamical information [6–8]. Therefore, we focus our efforts on performing cluster analysis for grouping MD conformations with high affinity in their substrate-binding cavities in order to extract the most relevant information during the molecular docking simulations, reducing its overall computational cost. Even though clustering is the computational intelligence approach employed in this work, we note that several papers employ learning approaches for the domain of molecular dynamics, with goals such as predicting bioactivities of ligands to target proteins [9], drug classification [10, 11], and free energy of binding prediction [5, 12, 13].

Clustering is the most suitable computational intelligence technique for dividing MD conformations into structurally homogeneous groups and for quickly understanding the resulting sets [14]. In this approach, every MD conformation is divided into several groups by using a measure of similarity/dissimilarity. Clustering of MD conformations is especially useful for molecular docking simulations since it provides groups of similar receptor structures. MD conformations that are placed in the same group are, according to some criterion, similar to each other and dissimilar from the conformations of other groups [15]. Hence, if a receptor conformation belongs to a cluster that interacts favorably with a specific ligand, one could assume that other conformations within the same cluster will behave similarly. Otherwise, the conformations belonging to this cluster are considered unpromising and consequently may be discarded in order to reduce the number of docking experiments on the FFR model. This smaller model is called the reduced fully flexible receptor (RFFR) model [8]. wFReDoW [8] is a cloud-based web environment that efficiently generates RFFR models. It reduces the dimensionality of FFR models by performing the selection of the most promising clusters of structures during docking experiments. However, wFReDoW requires as input a clustering partition of MD structures and the better the partition, the better the final performance of wFReDoW.

The Root mean square deviation (RMSD) values obtained by pairwise or matrix error distances are the most traditional and popular measure of similarity used for partitioning MD trajectories. For instance, Lyman and Zuckerman [16] generated a set of reference structures by enforcing a cutoff radius in RMSD for cluster assignment from biomolecular simulation trajectories of metenkephalin, a pentapeptide neurotransmitter. Shao et al. [17] make use of several clustering algorithms and two validity metrics to find the best clustering partition based on the pairwise RMSD values of small samples from an MD trajectory. Even though the meaningful trajectories cover very different portions of the conformation space, they limited the structural metrics by using only a portion of the data, and then the remaining data were added to existing clusters. Torda and van Gunsteren [18] were interested in algorithms which did not require previous selection of cutoffs for cluster size or the number of clusters, with the goal of naturally creating clusters based on the DME (*D*_*ab*_) over all pairs of atoms being considered in the structural configuration.

In this paper, we aim to identify relevant conformational changes that occur into the substrate-binding cavity along an MD simulation trajectory for reducing the dimension of the FFR model during docking experiments. For this reason, we are interested in investigating the (dis)similarities in a specific site or cavity, rather than taking into account the motions that occur within the entire MD structure as it is the case when using the RMSD deviation. For such case, we generate and analyze partitions based on features from the binding cavity of an MD simulation regarding the InhA-NADH complex [19] through the well-known *k*-means algorithm [20]. To the best of our knowledge, this is the first approach that employs properties from the substrate-binding cavity of every MD structure in order to measure similarity among trajectories. The quality of the resulting data partitions were evaluated according to three clustering validity criteria, namely, the Davies-Bouldin (DB) index [21], Dunn's index [22], and the gap statistic [23]. To validate whether the selected partition generated groups of structures that share similar conformation features, we analyzed the distribution of the free energy of binding (FEB) values that are generated after performing exhaustive docking experiments between the 20,000 conformations of the FFR model and 20 different ligands on AutoDock4.2 [24]. The best selected partition is used as input to the wFReDoW environment [8] in order to considerably reduce the time taken for the drug discovery process, as well as providing a more accurate ranking of potential drugs for the FFR model under study.

This paper is organized as follows.  Section 2 shows the structural features extracted from the substrate cavity for clustering the MD simulation trajectory, as well as the clustering validity criteria and the 20 compounds used to conduct the experimental evaluation. The cluster analysis and validation are presented in Section 3 along with a discussion on our findings. Finally, Section 4 presents our conclusions and opportunities for future work.

## 2. Materials and Methods

### 2.1. Data Set for Clustering the MD Trajectory

To generate an RFFR model to be used as input to wFReDoW, conformational features from the substrate-binding cavity were employed during the clustering process, which was performed by the well-known *k*-means algorithm [20]. *k*-means is a widely used clustering algorithm that has been recently applied on MD trajectory studies [17, 25, 26]. It is a hard-partitioning-based strategy that attempts to find a user-defined number of clusters (*k*) by locally optimizing the average squared distance of objects from their nearest cluster center (centroid). Briefly, the *k*-means algorithm randomly generates *k* centroids and refines them through several expectation-maximization iterations, in which the cluster memberships are determined by computing the distance of every object to each of the *k* centroids [15].

In this work, we make use of a 20 ns MD simulation trajectory of the InhA-NADH enzyme complex from* Mycobacterium tuberculosis* (PDB ID: 1ENY) as described in [19]. Data for the MD ensemble were collected at every 1 ps, resulting in a set of 20,000 instantaneous receptor conformations. The 20 ns MD trajectory constitutes the FFR model employed as a case study to guide our research. The structural properties that were extracted from the substrate-binding cavity of every MD conformation are

1. the accessible surface area (in Å^2^),
2. the volume (in Å^3^),
3. the number of heavy atoms in the substrate-binding cavity of the enzyme (PDB ID: 1BVR) [27],
4. the pairwise RMSD distances between binding cavity atoms (in Å).

The first three properties were collected using the CASTp software (Computed Atlas of Surface Topography of proteins) [28]. CASTp provides an online resource for locating, delineating, and measuring concave surface regions on three-dimensional structures of proteins based on the solvent-accessible surface area model [29] and the molecular surface model [30]. The measurement of surface area and volume for every MD conformation was obtained by considering the residues that enclose the cavity of the InhA substrate analog from the 1BVR structure [27], which contains the largest number of atoms.

**Figure 1:**
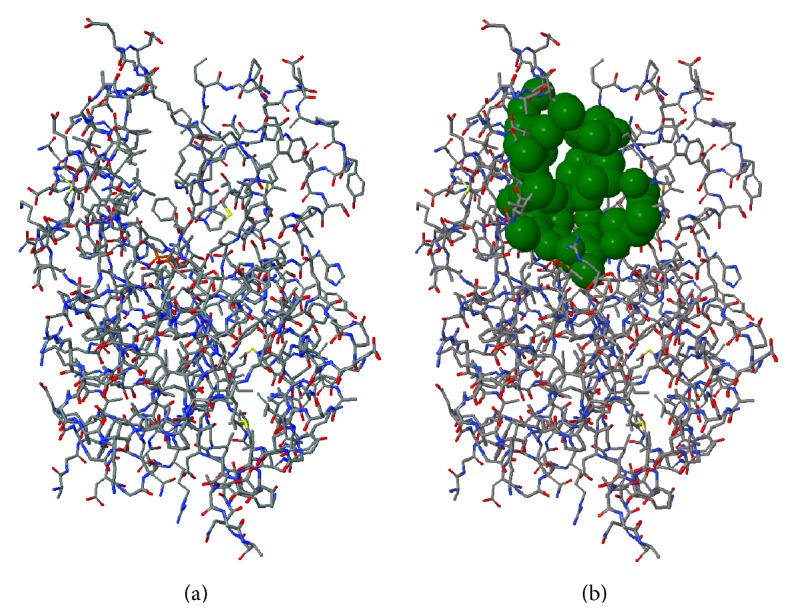
Substrate-binding cavity of the InhA enzyme from* Mycobacterium tuberculosis* (PDB ID: 1BVR) identified by the CASTp software. The stick representation is colored by atom type (carbon and hydrogen: light grey; nitrogen: blue; oxygen: red; sulphur: yellow). (a) Chain A of the 1BVR crystal structure submitted to CASTp. (b) In green the substrate-binding cavity of the 1BVR enzyme represented by van der Waals spheres.

Figure 1 shows the substrate-binding cavity of the 1BVR enzyme collected by the CASTp software. The pairwise RMSD distances were evaluated by monitoring the differences between the backbone atoms (N, C*α*, C, and O) within the substrate-binding cavity from the first structure against the conformation under comparison. The RMSD values were calculated using the* ptraj* module from AmberTools12 [31].

With this dataset, we seek to cluster different behaviors found within the substrate-binding cavity along an MD simulation, which in turn may help identifying which of the clusters contain snapshots that interact more favorably with a specific compound during the wFReDoW docking experiments. It is worth mentioning that this methodology is not specific to a single protein; it may also be used for other enzymes, as long as their binding pockets are known in advance.

### 2.2. Clustering Validity Criteria

The criteria employed to evaluate the quality of the generated partitions are the Davies-Bouldin index [21], Dunn's index [22], and the gap statistic [23]. These measures have been shown to be interesting strategies for evaluating the quality of clustering partitions, especially when using them together with a further manual examination of the generated clusters [17].

The Davies-Bouldin (DB) criterion is based on the ratio of within-to-between cluster distances. It is defined as

(1) $$\begin{array}{c} \text{DB}=\frac{1}{k}\overset{k}{\underset{i=1}{\sum }}\underset{j\neq i}{\max}\left\{D_{i,j}\right\},\;D_{i,j}=\frac{{\bar{d}}_{i}+{\bar{d}}_{j}}{d_{i,j}}, \end{array}$$

where *k* is the number of clusters, *D*_*i*,*j*_ is the within-to-between cluster distance of the *i*th and *j*th clusters, ${\bar{d}}_{i}$ and ${\bar{d}}_{j}$ are the average distance between each object in the cluster with the respective centroid, and *d*_*i*,*j*_ is the distance between centroids of the *i*th and *j*th clusters.

Similarly to DB, Dunn's index [32] also indicates the best partitions based on geometrical considerations regarding large distances between clusters and compactness within cluster. Partitions that comprise compact and well-separated clusters are assigned large values of Dunn's index, as indicated in the following equation:

(2) $$\begin{array}{c} D_{n}=\underset{1⩽i⩽n}{\min}\left\{\underset{1⩽j⩽n,j\neq i}{\min}\left\{\frac{\delta \left(C_{i},C_{j}\right)}{\max_{1⩽k⩽n}\mathrm{diam}\left(C_{k}\right)}\right\}\right\}, \end{array}$$

where *δ*(*C*_*i*_, *C*_*j*_) is the set of the intercluster distance between clusters *C*_*i*_ and *C*_*j*_, and diam⁡(*C*_*k*_) is the intracluster diameter of the *k*th cluster.

The gap statistic [23] is based on a comparison of the within-cluster sum of squared distances of the given partition with a partition obtained from random data. It is a powerful procedure for estimating the number of cluster for a dataset, which compares the changes in the within-cluster dispersion with that expected under an appropriate null distribution used as reference, as follows:

(3) Gapnk=En∗log⁡Wk−log⁡WkWk=∑r=1k12nrDr,

where *n* is the sample size, *k* is the number of clusters being evaluated, *W*_*k*_ is the pooled within-cluster dispersion, *n*_*r*_ is the number of data objects in cluster *r*, and *D*_*r*_ is the sum of the pairwise distances for all objects in cluster *r*. The gap statistic is calculated for partitions with varying *k*, and the highest value within a tolerance range is considered the optimal *k*.

Whereas DB and Dunn's index aim at identifying partitions that are compact and well-separated, the gap statistic tends to estimate the optimal number of clusters based on the dispersion of the clusters. An optimal partition should provide a high value for Dunn's index and the gap statistic and a small value for DB.

### 2.3. Clustering Validation Methodology

**Figure 2:**
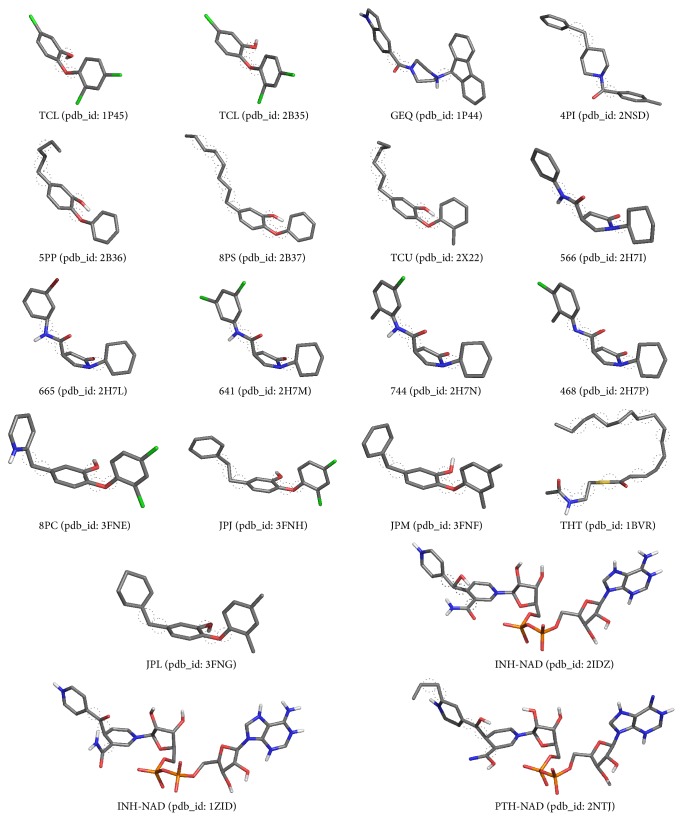
Stick representation of the 3D structures of the 20 ligands used in docking experiments. Each ligand, with its structures colored by atom type, is identified by their name and their corresponding PDB identification (PDB ID). The dashed circle represents the rotatable bonds selected by AutoDockTools 1.5.6.

After defining the optimal partition through the clustering validity criteria, we perform exhaustive docking experiments on AutoDock4.2 with the intention of searching for evidence that validates the quality of such a partition. These experiments are conducted between 20,000 snapshots (FFR model) and 20 different compounds, which are extracted from 20 InhA structures deposited at PDB [33].  Figure 2 shows the 3D structures of the 20 compounds and the rotatable bonds defined in the docking experiments.

We build the FFR model in this study from a 20 ns MD simulation of the InhA-NADH complex from* Mycobacterium tuberculosis* (PDB ID: 1ENY) [19]. In order to preserve the reaction mechanism between ligands and the target protein, NADH should be treated as a coenzyme. Hence, for experiments with ligands, the coenzyme was considered as part of the protein receptor structure. Conversely, we removed the NADH coenzyme from all snapshots of the FFR model when we performed the experiments with adducts (INH-NAD and PTH-NAD), since they already have the coenzyme as part of their structures.

The experiments were divided into two steps. In the first step, we identify the best *k* value for *k*-means clustering based on Dunn's index, DB, and the gap statistic. In the second step, we perform exhaustive docking experiments on the FFR model and 20 different compounds to validate the best clustering solution. Additionally, we analyze the accuracy, comprehensibility, and biological significance of the docking results.

## 3. Results and Discussion

As previously discussed, employing FFR models and databases of small compounds, such as GDB-17 [34], which holds more than 166 billion of compounds to perform practical virtual screening, often becomes an unfeasible task. The limiting factor that is present in this approach is the computational capacity of generating FFR models that sample longer time scales [35, 36].

Hence, the hypothesis we attempt to validate in this paper is that the proposed methodology for clustering the MD trajectory is capable of effectively identifying clusters of promising snapshots for specific ligands. More specifically, we investigate the problem of using FFR models to perform docking experiments for a set of compounds. One way to address this issue is to reduce the dimension of FFR models by selecting a representative sample of promising snapshots for each compound, preserving the essential structural properties of the model. With this in mind, we evaluate whether making use of clustering algorithms can help us to find out relationships between the interactions of FFR models and compounds. We concentrate efforts on using the *k*-means algorithm and analyze the result partition to verify our working hypothesis.

### 3.1. Cluster Analysis of the FFR Model

This section focuses on the execution of the *k*-means algorithm for clustering the MD trajectory in different numbers of clusters and then identifying the optimal partition according to the clustering validity criteria. This procedure is divided into three steps. First, we created the input dataset for the clustering algorithm. Then, we executed the *k*-means algorithm, with *k* ranging from 2 to 15 centroids. Finally, we identified the suitable MD clustering by using DB [21], Dunn's index [22], and the gap statistic [23].

**Figure 3:**
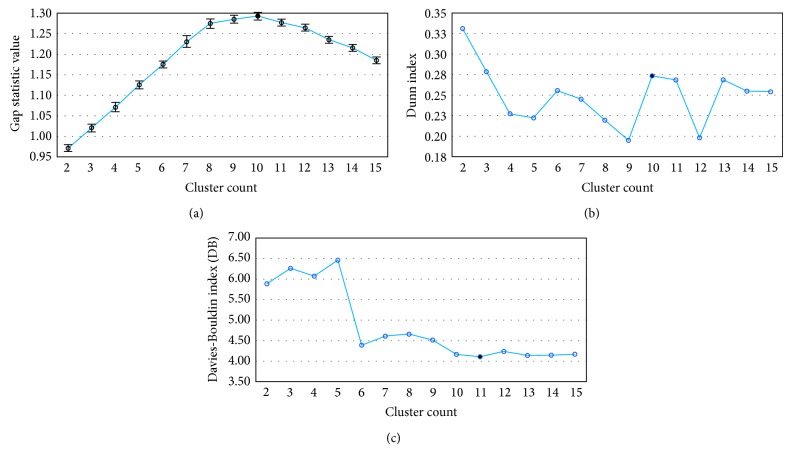
Clustering validity criteria for the MD trajectory of the InhA enzyme as a function of the number of clusters. (a) Gap statistic. (b) Dunn's index. (c) DB index. Black circles identify the best number of clusters for each criterion. The best gap result was used as the decisive criterion for selecting between *k* = 10 and *k* = 11, as suggested by Dunn's index and DB, respectively.

As described in Section 2.1, we extracted the structural properties from the substrate-cavity binding of each receptor conformation that makes up the FFR model. The area, volume, RMSD, and the score of heavy atoms for the 20,000 conformations were placed in a CSV file. As our dataset comprises attributes with different units and scales, we normalized all values before executing the *k*-means algorithm. The numeric values were normalized to lie in a scale within the interval [0,1]. The CSV file with the normalized data was submitted to the *k*-means algorithm and it was executed with *k* ranging from 2 to 15 clusters (Figure 3). This range of values was defined based on the wFReDoW environment, which creates balanced queues of tasks taking into account the number of clusters and HPC workstations allocated for the docking experiments. De Paris et al. [8] concluded that the best wFReDoW performance is obtained when only 30% of the snapshots have been docked. For that reason, we decided that a potential solution is to insert at least two receptor structures of each cluster in the queues of tasks created by the wFReDoW environment.

In order to evaluate the quality of the *k*-means partitions and to identify the best solution, we calculated the gap, DB, and Dunn's values for the partitions with distinct numbers of clusters. Partitions that provide a low value for DB and high value for gap and Dunn's values suggest better clustering. Note by observing Figures 3(a) and 3(b) that *k*-means generates a partition that shows a clear maximum value for gap and Dunn's values when clustering data into 10 clusters. Nevertheless, DB (Figure 3(c)) shows a slight preference for 11 clusters instead of 10.

Note that Dunn's index also indicates that the partition with two clusters is a good solution. However, this same partition is poorly evaluated by DB and the gap statistic. The latter was used as the decisive criterion for the two partitions suggested as optimal by DB, that is, the partitions with 10 and 11 clusters. Following this strategy, we selected the partition with 10 clusters considering that its gap and Dunn's values are higher than the partition with 11 clusters.

**Figure 4:**
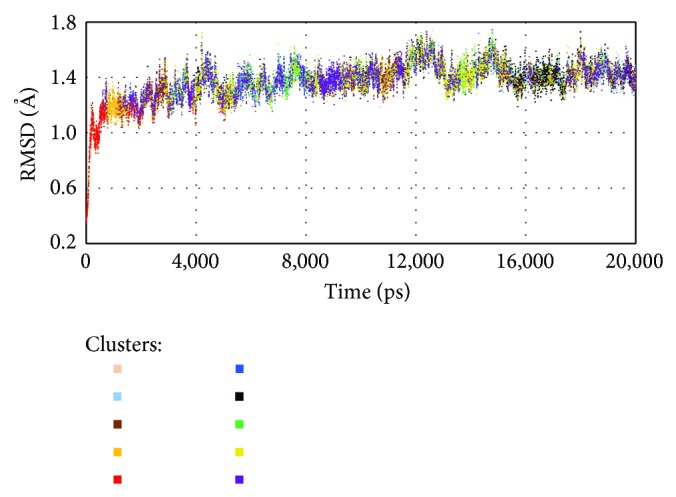
Cluster distribution along the InhA enzyme MD trajectory from the optimal *k*-means partition. Each object represents different backbone (N, C*α*, C, and O) RMSD values as a function of time over the trajectory which are colored based on their cluster memberships.

To illustrate the optimal *k*-means partition, we present in Figure 4 the effects of clustering different timescales in the MD trajectory based on its structural features. As we might have expected, the clustering outcome is strongly influenced by the structural changes on the substrate-binding cavity to determine the similarity/dissimilarity of the different molecular configuration. In particular, contrary to those methods based only on RMSD data where the clustering tends to show strips along the MD trajectory [17, 18], the proposed methodology shows a heterogeneous distribution of the clusters.

We have also performed several attempts to discover relationships between pairwise RMSD distances and FEB values from the MD structures under study. However, no satisfactory relationship was noticeable between them, probably due to the fact that RMSD abstracts all important features that greatly influence the FEB value. The distribution in Figure 4 depicts the identification of similar cavities in different timescales along the MD simulation. In the next section, we perform a case study that provides several insights regarding the relationship between the MD clustering distribution and the FEB values.

### 3.2. Validating the Optimal Data Partition

The purpose of performing this last set of experiments is to examine similarity patterns among the clusters of MD structures when they are submitted to molecular docking simulations with a set of different ligands. With this case study, we expect to identify behaviors that are directly related to the attributes used to cluster the FFR model. Since attributes are based on structural features of the substrate-binding cavity of each snapshot that makes up the FFR model, we intend to seek the snapshots belonging to a cluster that interacts

1. favorably with a specific ligand, but adversely with other ligands,
2. adversely with a specific ligand, but favorably with other ligands,
3. favorably with a set of ligands,
4. adversely with a set of ligands.

Unlike other studies, which select a set of representative snapshots with dissimilar RMSD distances in MD trajectories, we concentrate efforts on partitioning snapshots according to a level of affinity in their binding cavity, aiming at identifying promising snapshots during the virtual screening of ligands. We focus on providing evidences that the proposed partition is capable of covering a set of compounds experimentally tested through analyses on the docking results. For such case, we performed molecular docking simulations between the FFR model and 20 compounds (Figure 2) using FReMI (Flexible Receptor Middleware) [8]. FReMI, which is part of the wFReDoW environment, is a middleware developed to execute exhaustive docking experiments of FFR models with maximum efficiency through multiprocessing machines with AutoDock4.2 [24].

For this experiment, the Lamarckian genetic algorithm (LGA) from AutoDock4.2.5.1 was used to execute the molecular docking experiments. The maximum number of energy evaluations and the number of runs were set to 300,000 and 25, respectively. The grid box dimensions were tailored according to each ligand type. We also defined the atom types of AutoDock4.2, added the Gasteiger charges, and merged the nonpolar atoms for each snapshot of the FFR model. The rotatable bonds highlighted for each ligand in Figure 2 were applied to execute the docking experiments.

**Figure 5:**
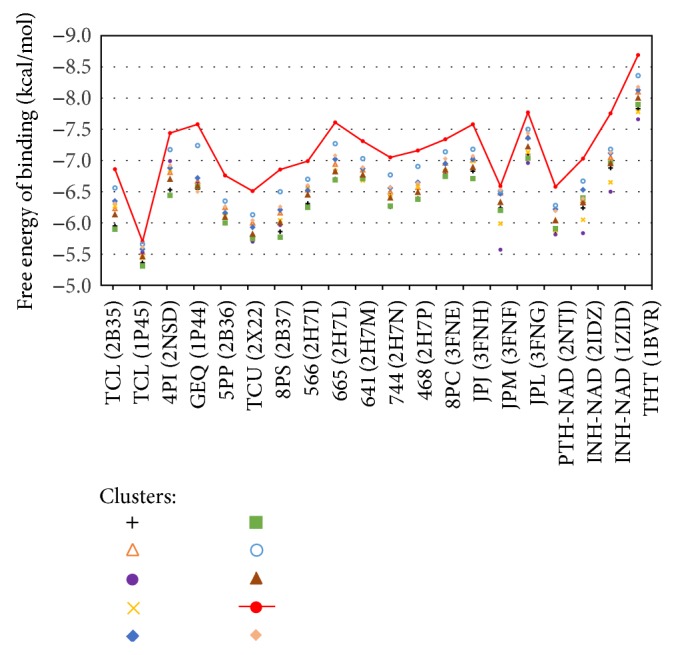
Evaluation of median FEB values of clusters as a function of compounds. The red circle represents the cluster with best median FEB values for each experiment. The red line highlights the clusters with the best median FEB values at the top.

In order to validate the optimal clustering solution from the docking results, we analyzed the variance among FEB values obtained in the clusters for each ligand, separately. We first extracted all FEB values from the docking experiments. Then, we linked the snapshots with their respective clusters. Finally, we calculated the median FEB values of the clusters independently for each compound. To illustrate this assessment, Figure 5 shows the variation in the median FEB values of the 10 clusters as a function of ligands. From the docking results it is possible to see potential behaviors, which in turn validate the proposed methodology of reducing the FFR model. For instance, the red line indicates the best median FEB values in the same cluster for all ligands tested. This confirms that the selected partition is capable of detecting a similarity pattern for representing the best results of these set of ligands.

An important finding shown in Figure 5 is that all ligands present their best median FEB values in the same cluster (see the red line). Further, this best cluster appears to be well-separated compared to the remaining clusters. This is useful if one desires to considerably reduce the time to perform docking experiments on the FFR model. However, it is unsafe to generalize that one cluster will obtain equal behavior for libraries of small molecules merely based on the docking results of 20 ligands. An optional strategy to reduce the time for virtual screening of large libraries is to dynamically perform a selection of those clusters that contain the most promising interactions during the molecular docking simulations, as it is deployed on wFReDoW. Thus, the structural changes of different ligands may be detected more accurately for the induced-fit effects and for ensembles of reduced and representative MD structures; that is, the RFFR models will be tailored to a specific ligand.

Comparing the FEB values, Figure 5 indicates that the clusters contain a similar sort of sequence with respect to the median FEB values for all ligands. We observed that the similar structures presented in Figure 2 also have similar median FEB values in the docking experiments with the FFR model. For instance, Figure 5 shows equal behavior for compounds JPJ (3FNH), JPM (3FNF), and 8PC (3FNE) since they only vary in their spatial arrangements. This is surprising because it was expected that the clusters should vary from one compound to another as we described at the beginning of this section. Although this was not expected, it is considered a positive indicator to validate our approach. The proposed methodology is capable of preserving a standard behavior for all compounds tested by clustering snapshots that contain high structural similarity in their substrate-binding cavity.

## 4. Conclusions and Future Work

This work proposes a computational intelligence-based methodology that employs a clustering algorithm to analyze an MD trajectory of the InhA enzyme. The proposed approach employs clustering validity criteria to find out the optimal data partition for reducing the computational cost of molecular docking experiments.

Docking experiments on the FFR model were performed for 20 different ligands with the intention of validating the proposed methodology. Based on the docking results, we conclude that the generated data partition appears to successfully separate conformations regarding their FEB values, and a standard behavior is verified over all the ligands that were tested. The case study also shows that to consider the structural properties in the substrate-binding cavity as input for the clustering algorithm is a promising approach for clustering an MD trajectory of the InhA enzyme.

In comparison with other studies that propose to cluster MD trajectories, the methodology proposed in this paper has some essential advantages: it separates the clusters based on a set of structural properties from the substrate-binding cavity and it effectively identifies the protein structural changes in the target cavity. Besides using clustering validity criteria to select the optimal data partition, we further validate the proposed methodology to verify our hypotheses with regard to clustering MD trajectories. The limitation of the proposed methodology is that it is highly dependent on the prior knowledge on the target cavity for the protein under study.

The increasing availability of computing power is allowing longer timescales simulations [36]. Due to this progress, novel and promising computational techniques that address the problem of efficiently sampling receptor MD conformations should be investigated and tested. Our research shows the advantages of clustering MD trajectories for receptor proteins with a well-defined target cavity. As for future work, we intend to empirically analyze the performance of other clustering algorithms, such as hierarchical and SOM algorithms, to assess if there are significant differences with the findings reported in this study. Moreover, we intend to make use of even larger MD trajectories towards experimentally validating the methodology proposed in this paper.
